# Evaluation of side effects and compliance to chemotherapy in breast cancer patients at a Nigerian tertiary hospital

**DOI:** 10.3332/ecancer.2023.1537

**Published:** 2023-04-21

**Authors:** Sharif Adeniyi Folorunso, Oyindamola Olajumoke Abiodun, Abbas Adesina Abdus-Salam, Funmilola Olanike Wuraola

**Affiliations:** 1 Department of Radiology, Obafemi Awolowo University Teaching Hospital, Ile Ife, Nigeria 220101; 2 Department of Pharmacology and Therapeutics, University of Ibadan, Ibadan, Nigeria 200005; 3 Department of Radiation Oncology, University College Hospital/University of Ibadan, Ibadan, Nigeria 200005; 4 Department of Surgery, Obafemi Awolowo University/Obafemi Awolowo University Teaching Hospitals Complex, Ile Ife, Nigeria 220101

**Keywords:** breast cancer, chemotherapy, side effects, compliance

## Abstract

**Background:**

Chemotherapy improves tumour control and survival, but it may be associated with side effects (SEs) which can impair treatment compliance and worsen outcomes. Assessment of patients in routine clinical practice, outside clinical trials, may provide the information on effects of chemotherapy on patients and its impacts on treatment compliance.

**Aim:**

To assess the SE and compliance to chemotherapy in breast cancer patients.

**Methodology:**

A prospective study involving 120 breast cancer patients receiving chemotherapy was carried out at the oncology clinics of the University College Hospital Ibadan. SEs reported were recorded and graded using Common Toxicity Criteria for Adverse Events version 5. Compliance was defined as a receipt of planned cycles of chemotherapy in the planned doses within the planned duration. The data collected were analysed using the Statistical Package for the Social Sciences software version 25.

**Results:**

The patients were all females with a mean age of 51.2 ± 11.8 years. Patients reported between 2 and 13 SE with a median of 8 SE. Forty-two (35.0%) missed at least one course of chemotherapy while 78 (65%) were compliant. The reasons for non-compliance were deranged blood test 17 (14.2%), chemotherapy SE symptoms related 11 (9.1%), financial constraints 10 (8.3%), disease progression 2 (1.7%) and transportation-related 2 (1.7%).

**Conclusion:**

Breast cancer patients encounter multiple SEs from chemotherapy which led to non-compliance with the treatment. Early identification and prompt treatment of these SEs will improve compliance with chemotherapy.

## Introduction

Globally breast cancer incidence is rising with estimated 2,261,419 new cases of breast cancer and 684,996 deaths resulting from the disease worldwide in 2020 [1]. Breast cancer is the most common female malignancy in Nigeria, with age-standardised incidence rate of 54.4/100,000

[2]. In Nigeria, like other low-middle-income countries, the incidence rate is low compared to high-income countries but it records a high mortality rate. This is due to late presentation, inadequate treatment and other barriers [3]. The higher incidence in developed countries may be due to higher life expectancy, increasing obesity, reduction in fertility rates and rising age for first births [4]. Treatment options for breast cancer include local treatment like surgery and radiotherapy as well as systemic treatment such as chemotherapy, hormonal therapy and targeted therapy. Chemotherapy improves tumour control, increases the chance of cure and prolongs the lives of breast cancer patients [5]. About 80% of breast cancer cases in Nigeria will present at stage III/IV also approximately 40% of patients with breast cancer are triple negative [3]. These patient groups will require systemic therapy.

The choice of chemotherapy regimen for breast cancer has evolved over the years. Previously, cyclophosphamide, methotrexate and 5 fluorouracil (CMF) were the gold standards [6]. Clinical trials in the 1990s, however, showed that anthracycline-based chemotherapy (ABC) (usually a combination of epirubicin or adriamycin with cyclophosphamide) showed superior benefits [7, 8]. More recently, trials have shown taxane (notably docetaxel) or platinum can further improve survival, especially for triple-negative breast cancer [9]. Current treatment guidelines recommend ABC or taxane-based chemotherapy (TBC) as the preferred regimen for breast cancer [5, 10]. In Nigeria, ABC is the most frequently prescribed chemotherapy for breast cancer possibly because it is relatively cheaper as most patients pay out of pocket [11].

Chemotherapeutic agents exert their cytotoxic effects by disrupting the processes in the cell cycle [12]. Cancer cells are rapidly dividing which makes them susceptible to chemotherapy [13]. Rapidly dividing normal cells could also be affected in the process leading to toxicities [14]. Although these agents affect both normal and cancer cells, their therapeutic potential stems from their ability to cause greater damage in cancer cells as opposed to normal cells when received as scheduled [12]. Non-compliance to the schedule can reverse this trend leading to accelerated repopulation of the cancer cells and worsening the outcomes.

Gastrointestinal disorders, bone marrow suppression, neuropathies, hair loss, fatigue and skin disorders are side effects (SEs) often reported by cancer patients [5, 9]. Chemotherapy was described as the most unpleasant cancer treatment and the fear of its SEs can lead to late presentation for cancer treatment [15]. Prompt assessment and treatment of these SEs will improve treatment compliance and reduce hospital admission or treatment discontinuation [14, 16]. Knowledge about SEs often comes from clinical trials which might not really reflect the reality of the SEs in clinical practice as patients with high risk are often excluded from trials, and safety monitoring is more intensive than during routine care [17]. Pragmatic data collection will provide information about the real exp**e**rience of cancer patients as regards chemotherapy SEs. Previous studies in Nigeria have tried to assess SEs of chemotherapy and their burden on breast cancer patients; however, it was carried out when CMF was the mainstay of treatment [18]. Studies conducted later included all other malignancies [19, 20]. The major limitation of these studies was the wide diversity in the chemotherapy regime used. Each cancer has its own clinical course and is managed with different chemotherapy which influences the SEs experienced by patients. This study aims to assess the SEs and treatment compliance to the chemotherapy regimen for breast cancer.

## Methodology

This is a prospective study carried out at the Radiation and Surgical Outpatient Clinics of the University College Hospital, South West Nigeria. The study was conducted for 6 months between June 2021 to December 2021. The study population includes patients with histologically diagnosed breast cancer scheduled to receive chemotherapy at the clinics. Newly diagnosed breast cancer patients and patients with disease recurrence following the previous treatment, receiving either ABC or TBC for breast cancer were included. Patients who switched over from one chemotherapy regimen to another, patients with WHO performance status >2, patients receiving radiotherapy concurrently with chemotherapy and patients who were diagnosed with another type of cancer were excluded from the study. Subsequently, individuals who met these criteria were invited to take part in the study.

### Sample size determination

For populations less than 10,000, this formula is used: $n\;f\;=\;\frac{n}{1+n/N}$

where *n* is the sample size for populations greater than 10,000 people which is calculated using the formula developed by Cochran as: *n* = *Z*^2^ *p q*/*d*^2^

*Z* = confidence interval is 1.96

*p* = prevalence (proportion in target population estimated to have the particular characteristic). Average prevalence of chemotherapy SEs is 0.54 [17].

*q* = 1−*p* = 1−0.54 = 0.46

*D* = precision value is 0.05

*n* is therefore $\frac{{(1.96)}^{2}\times 0.54\times 0.46}{{(0.05)}^{2}}=382$

For populations less than 10,000, this formula is used: $n\;f\;=\;\frac{n}{1+n/N}$

where *n* (sample size for the population greater than 10,000)

*N* = study population = 154 (approximate number of women with breast cancer seen in 6 months)

*n f* = 384 ÷ (1 + 384/154) = 384 ÷ (1 + 2.47) = 110.03

A sample size of 120 participants was selected.

### Data collection procedure

One hundred and twenty consecutive breast cancer patients who met the inclusion criteria and gave consent were invited to participate. The study was explained to them and informed consent forms were signed by the patients. The primary data collection included a review of medical records together with patient interviews. Data retrieved from the medical record included patients’ sociodemographic, clinical and treatment information. Patients received chemotherapy in courses usually 3 weeks apart. They were followed up for the first three courses for SEs and compliance with the chemotherapy schedule. Data on chemotherapy SEs were completed before receiving the fourth course of chemotherapy.

Interviewer administered questionnaire was used with items developed from reviewing relevant literature [6, 17, 21]. A pilot test of the instruments was performed among 15 patients with breast cancer in the Radiation Oncology Department, University College Hospital Ibadan (UCH), Ibadan, to determine the reliability, acceptability and clarity of the questionnaire items. The test on 15 items of chemotherapy SEs grading yielded a Cronbach’s alpha of 0.80.

Chemotherapy SEs that are often reported in literature and easy to recognise by patients were included. The SEs reported were graded with the National Cancer Institute Common Toxicity Criteria for Adverse Events version 5. Grade 1 was defined as mild, grade 2 as moderate, grade 3 as severe and grade 4 was life-threatening [23]. Organ systems included were gastrointestinal, general disorders and administration site conditions, nervous system, skin and subcutaneous tissue disorders and urinary disorders. There was space for SEs reported but not included in the developed list.

### Data management and analysis

Statistical Package for Social Sciences version 25 was used to analyse the collected data. The sociodemographic data, patients’ clinical characteristics, frequency and grading of the SEs were presented in frequency distribution tables. The number of SEs reported was compared between groups of chemotherapy regimes using Mann–Whitney test. The proportions were compared using the chi-square test. The significance level was set at less than 5%.

### Ethical approval

Approval was obtained with registration number UI/EC/21/0289 from the joint institutional ethical review committee of the University of Ibadan and the University College Hospital, Ibadan.

## Results

A total of 120 patients who met the selection criteria took part in this study. The sociodemographic, clinical and treatment information of study participants is presented in Table 1. The ages ranged from 22 to 78, with an average of 51.2 years. Sixty-seven (55.8%) patients were in the 41–59-year age category, 25 (20.8%) patients were younger and 28 (23.3%) patients were older. All the patients were female. Most of the cases seen were stage III, 76 (63.3%) followed by stage IV, 28 (23.3%) and stage II 14 (11.7%) (Table 1).

Most of the patients 104 (86.6%) received dual agents, 7(5.8%) were on triple regimen while 9 (7.5%) were on single agents. Of the two-chemotherapy regimen prescribed for breast cancer patients, anthracycline-based is the commonest 82 (68.3%) (Table 1). Epirubicin with cyclophosphamide was the most common ABC prescribed while paclitaxel with platinum was the most common TBC administered (Table 1).

**Figure 1.**
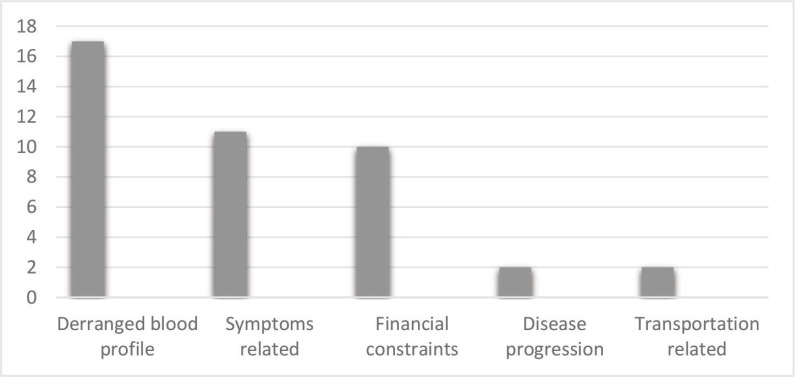
Reasons for non-compliance to chemotherapy.

Patients reported between 2 and 13 SEs in the course of their chemotherapy with a median of 8 SEs. A total of 913 SEs was reported by the 120 patients, of which 610 (66.8%) were mild, 292 (32.0%) were moderate and 7 (0.8%) were severe. None of the patients reported life-threatening SEs (Figure 1). The following SEs were most commonly reported: fatigue 113 (94.2%), alopecia 113 (94.2%), loss of appetite 99 (82.5%), nausea 93 (77.5%), nail discolouration 93 (77.5%), skin hyperpigmentation 75 (62.5%) and headache 60 (54.5%), (Table 2). Very few patients had severe SE such as diarrhoea 2 (1.6%), loss of appetite 2 (1.6%), nausea 1 (0.8%), malaise 1 (0.8%) and vomiting 1 (0.8%) (Table 2).

The frequency of SEs in patients that received ABC and TBC was analysed. Patients on ABC reported significantly higher diarrhoea (*p* = 0.039), vomiting (*p* = 0.020), headache (*p* = 0.006), nail discolouration (*p* = 0.010) and skin hyperpigmentation (*p* = 0.020) than the patients on TBC (Table 3). The median SEs reported by each patient were also significantly higher in patients on ABC than in patients on TBC, *p* = 0.044 (Table 3).

Close to two-thirds, 78 (65%) received the chemotherapy as planned (compliance), while 42 (35.0%) had disruption or delay in scheduled chemotherapy (non-compliance). The reason for non-compliance was deranged full blood counts 17 (14.2%), chemotherapy SE symptoms related 11 (9.1%), financial constraints 10 (8.3%), disease progression 2 (1.7%) and transportation-related 2 (1.7%) (Figure 1).

## Discussion

A total of 120 breast cancer patients were recruited to participate in this study. The average age was 50.45 years, with the 40–59 age group in the majority. This study showed that breast cancer occurs primarily in the young and middle-aged groups in this environment. This is similar to the findings of other studies in Nigeria which showed that breast cancer occurs at a younger age compared to Caucasians [4, 24–26]. People in this age group are at the productive age.

All the patients recruited in this study were female. This differs from the findings of a previous study in Lagos that reported 98% female and 2% male. The difference in the findings may be due to differences in the inclusion and exclusion criteria. While the study in Lagos recruited all breast cancer patients in the hospital, this study reviewed patients on chemotherapy and excluded patients on radiotherapy.

Most of the cases seen either have stage III (63.3%) or stage IV, 28 (23.3%). These stages connote advanced disease. Previous studies have demonstrated that cancer patients in Nigeria, often present with advanced diseases [25]. This may be due to aggressive tumour biology in blacks when compared with white [4]. Other possibilities for the late presentation as reported by a previous study are lack of awareness of cancer symptoms, seeking alternative care, fear of diagnosis and treatment and the challenges of distance to available centres of treatment [20].

In this study, the majority of the patients (87%) received combination chemotherapy. The recommended guideline for the treatment of breast cancer is generally the use of two or more agents simultaneously or consecutively. Combination therapy is known to have a higher objective response rate and a longer time to progress than a single agent [27, 28]. The most common chemotherapy regimen used was anthracycline-based. Anthracycline-containing regimen is one of the preferred chemotherapy regimens for breast cancer recommended by treatment guidelines [10]. It is also relatively cheaper than other preferred regimes which further favours its prescription [11]. This is particularly important as most patients in the country pay at their own expense.

Each of the patients reported SEs ranging between 2 and 13, most of which were mild. This may be because the majority of the patients were on multiple agents. As a rule of combination therapy, the selected drug should have non-overlapping toxicity [29]. This allows toxicity to be distributed across multiple organ systems and avoids significant toxicity in individual organs [29]. Patients on ABC reported more SEs when compared with taxane-based (8 versus 7, *p* = 0.044). This is in agreement with the findings of Nyrop *et al* [16] (7 versus 5, *p* = 0.01).

The most commonly reported SEs by participants in this study was fatigue. This is in keeping with the findings of previous studies [16]. A survey of 4,600 cancer patients receiving chemotherapy showed the most frequent SEs was fatigue [30]. Another study revealed that the commonest SE experienced by breast cancer patients receiving chemotherapy in Lagos was nausea [18]. The difference in the findings may be due to the fact that the latter study used CMF-based chemotherapy which is hardly being used again in the treatment of breast cancer.

This study found that close to two-thirds of the patients had good compliance with chemotherapy. This is in keeping with the findings of a previous study in Enugu [31]. Higher compliance was demonstrated by previous studies conducted outside the country [16, 32]. The difference may be due to better health facilities in those countries that could manage chemotherapy SEs better and promptly. Also, out-of-pocket treatment financing of treatment is expected to affect compliance in Nigeria. The most common reasons for non-compliance in this study were chemotherapy SE symptoms related to deranged blood profile and financial constraints. More aggressive pre- and post-treatment medications can reduce the impacts of chemotherapy SEs. Prophylaxis erythropoietin or filgrastim can help reduce the frequency of deranged blood count profile and improve compliance [33] but this will come at an additional cost, especially if the patient will have to pay. Financial constraints are a major barrier to cancer treatment in Nigeria [20]. Health insurance should be expanded to cover all cancer patients in Nigeria.

## Limitation

Participants were recruited from a single institution which may not be a perfect representation of the population of breast cancer in Nigeria. A multicentre longitudinal study would have been more appropriate. Though all the patients received pre- and post-medications based on the institutional protocols, this study did not fully explore the pre- and post-treatment medications received by the patients. Nonetheless, our findings have provided insight into the experience of cancer patients as regards chemotherapy SE in routine clinical practice. These have also laid the foundation for future studies and interventions aim at improving treatment compliance and reducing the burden of chemotherapy.

## Conclusion

In this study, it was observed that an anthracycline-based regimen was the most commonly prescribed chemotherapy. Breast cancer patients on chemotherapy encountered multiple SEs from the chemotherapy. Close to two-thirds of patients were compliant with the chemotherapy as planned. The commonest reasons for non-compliance in this study were symptoms related to deranged blood profile and financial constraints.

## List of abbreviations

ABC: Anthracycline-based chemotherapy; CMF: Cyclophosphamide, methotrexate and 5 fluorouracil; SE: Side effects; TBC: Taxane-based chemotherapy; UCH: University College Hospital Ibadan.

## Ethics approval and consent to participate

Approval for this study was sought from the joint ethical review committee of the University of Ibadan/University College Hospital, Ibadan.

## Consent for publication

Consent was taken from the patients with aid of an informed consent form.

## Availability of data and material

The datasets used and/or analysed during the current study are available from the corresponding author upon reasonable request.

## Conflicts of Interest

None.

## Funding

None.

## Author contributions

SAF: Conceptualised the topic and designed the study methodology. He contributed to the data acquisition, data analysis, interpretation of data and wrote the final draft of this work.

OOA: Contributed to the conception, design, data acquisition, data analysis, interpretation of data and the draft of this work.

AAS: Contributed to the conception, design, data acquisition, data analysis, interpretation of data and the draft of this work.

FOW: Contributed to the conception, design, data acquisition, data analysis, interpretation of data and the draft of this work.

They have approved the submitted version and the final draft of the manuscript and take responsibility for this paper.

## Figures and Tables

**Table 1.** Study participants’ sociodemographic, clinical and treatment information.

Variables	Categories	*N* (%)
Age	≤40 years	25 (20.8%)
	41–59 years	67 (55.8%)
	≥60 years	28 (23.3%)
Sex	Female	120 (100%)
	Male	0 (0%)
Histology	Invasive ductal carcinoma	114 (95%)
	Others	6 (5%)
Stage	II	14 (11.7%)
	III	76 (63.3%)
	IV	28 (23.3%)
	Not specified	2 (1.7%)
Present chemotherapy regimen	**Anthracycline-based**	
	Adriamycin + Cyclophosphamide	22 (18.3%)
	Epirubicin + Cyclophosphamide	55 (45.8%)
	Epirubicin + Cyclophosphamide + 5 fluorouracil	5 (4.2%)
	**Taxane-based**	
	Docetaxel	9 (7.5%)
	Docetaxel + Platinum	10 (8.3%)
	Paclitaxel + Platinum	14 (11.7%)
	Paclitaxel + Cyclophosphamide	2 (1.7%)
	Docetaxel + Cyclophosphamide	1 (0.8%)
	Paclitaxel + Platinum + Cyclophosphamide	1 (0.8%)
	Docetaxel + Platinum + Cyclophosphamide	1 (0.8%)

**Table 2.** Frequency and severity of reported SEs.

Symptoms	Mild	Moderate	Severe	Total
Diarrhoea	27 (22.5%)	12 (10.0%)	2 (1.7%)	41
Oral mucositis	27 (22.5%)	5 (4.2%)	-	32
Loss of appetite	81 (67.5%)	16 (13.3%)	2 (1.7%)	99
Nausea	44 (36.7%)	48 (40%)	1 (0.8%)	93
Fatigue	77 (64.2%)	35 (29.2%)	1 (0.8%)	113
Vomiting	29 (24.2%)	23 (19.2%)	1 (0.8%)	53
Injection reaction	12 (10.0%)	-	-	12
Infusion reaction	6 (5.0%)	-	-	6
Headache	58 (58.3%)	2 (1.7%)	-	60
Alopecia	31 (25.8%)	82 (68.3%)	-	113
Nail discolouration	93 (77.5%)	-	-	93
Paraesthesia	52 (43.3%)	5 (4.2%)	-	57
Rash	11 (9.2%)	4 (3.3%)	-	15
Skin discolouration	12 (10.0%)	63 (52.3%)	-	75
Cystitis	33 (27.5%)	1 (0.8%)	-	34
Constipation	6 (5.0%)	-	-	6
Insomnia	7 (5.8%)	-	-	7
Dizziness	2 (1.6%)	-	-	2
Watery eye	2 (1.6%)	-	-	2
Total	**610**	**296**	**7**	**913**

**Table 3.** Comparison of frequency of SEs between patients taking ABC and TBC.

List of SEs	Anthracycline-based *N* = 82	Taxane-based *N* = 38	*p* value
diarrhoea	33 (40.2%)*	8 (21.1%)	0.039*
Oral mucositis	19 (23.2%)	13 (34.2%)	0.203
Loss of appetite	69 (84.1%)	30 (78.9%)	0.486
Nausea	64 (78.0%)	29 (52.6%)	0.833
Fatigue	78 (95.1%)	35 (92.1%)	0.512
Vomiting	44 (54.7%)	9 (23.7%)	0.020*
Injection reaction	9 (10.9%)	3 (7.9%)	0.601
Infusion reaction	4 (4.9%)	2 (5.3%)	0.928
Headache	48 (58.5%)	12 (31.6%)	0.006*
Alopecia	79 (96.3%)	35 (92.1%)	0.380
Nail discolouration	69 (84.1%)	24 (63.2%)	0.010*
Paraesthesia	35 (42.7%)	22 (57.9%)	0.121
Pruritus	6 (7.3%)	4 (10.5%)	0.554
Rash	9 (10.9%)	6 (15.8%)	0.458
Skin hyperpigmentation	57(69.5%)	18 (47.4%)	0.020*
Cystitis	20 (24.4%)	14 (36.8%)	0.159
Median number of SE reported by each patient (Median IQR)	8 (6, 8)	7 (5, 7)	0.044*

* Statistically significant

IQR: Interquartile range
